# No Children Should Be Left Behind During COVID-19 Pandemic: Description, Potential Reach, and Participants' Perspectives of a Project Through Radio and Letters to Promote Self-Regulatory Competences in Elementary School

**DOI:** 10.3389/fpsyg.2021.647708

**Published:** 2021-05-05

**Authors:** Jennifer Cunha, Cátia Silva, Ana Guimarães, Patrícia Sousa, Clara Vieira, Dulce Lopes, Pedro Rosário

**Affiliations:** Unidade de Investigação Aprendizagem, Instrução e Carreira, Centro de Investigação em Psicologia, Departamento de Psicologia Aplicada, Escola de Psicologia, Universidade Do Minho, Braga, Portugal

**Keywords:** COVID-19 pandemic, offline intervention, self-regulated learning, story-tool, radio, letters, elementary school, school closure

## Abstract

Around the world, many schools were closed as one of the measures to contain the spread of the COVID-19 pandemic. School closure brought about important challenges to the students' learning process. This context requires strong self-regulatory competences and agency for autonomous learning. Moreover, online remote learning was the main alternative response to classroom learning, which increased the inequalities between students with and without access to technological resources or for those with low digital literacy. All considered, to level the playing field for students without digital resources, there is an urgent need to promote self-regulatory competences through offline intervention solutions. The current paper describes a project with this purpose, using radio broadcasting and letters to reach elementary students without digital resources. Moreover, potential reach and participants' perspectives of the project implementation are presented and discussed. The project draws on a prior evidence-based story-tool intervention grounded on a self-regulated learning framework. The original intervention was set previous to the COVID-19 pandemic and was implemented in the classroom context (*N* = 1,103 students). Once the schools had been closed down, the mode of intervention was adapted with the collaboration of the community. Alternative solutions were developed as follows: (i) story chapters were read on the radio and students received to their homes a printed script, prompting reflection, and suggesting related activities; (ii) students were provided with the story-tool to read autonomously and received letters from the main characters of the story which included, for example, suggestions for activities and reflection. These two alternative modes of intervention delivery potentially reached 394 elementary students, including students with digital resources. Interviews conducted with a group of students were provided information about the positive aspects of these two modes of intervention delivery, perceived learning (e.g., planning), constraints, and suggestions to improve the project. The current work is likely to merit attention from researchers and educational practitioners, given the need to use offline alternatives to provide support for students without digital resources to engage in autonomous learning during the pandemic period. This project may also be used as an alternative or a complementary solution to online modality.

## Introduction

On the last day of 2019, a new coronavirus was potentially identified in Wuhan, China, according to the World Health Organization (2020). Rapidly, the virus spread to other countries, and 3 months later, the WHO classified COVID-19 as a pandemic. Meanwhile, around the world, several schools were closed or partially closed as a means to contain the spread of the virus (Viner et al., 2020). According to the data provided by UNESCO (2020), by April 2nd, 84.8% (about 1.5 billion) of students (considering all school levels) were affected by this health policy. The duration and extension of this policy in the school year of 2019–20 varied widely among the countries (from 2 weeks to 3 months) (Reimers and Schleicher, 2020). Focusing on the Portuguese case, schools were closed for more than one school term (i.e., more than 3 months).

As a response to the school closure, governments all over the world provided different solutions to ensure learning and instruction continuity, online, and/or broadcast remote learning being among the available modes mostly offered (Reimers and Schleicher, 2020). The latest UNICEF report on remote learning policies during the pandemic provides data based on more than 110 countries (UNICEF, 2020). For all educational levels, the digital mode to deliver learning contents, requiring computer, and internet access, were the most used, followed by broadcast responses using television, and then radio. However, in low-income countries, educational stakeholders used mainly the radio broadcasting to reach students. Surprisingly, there are no results about paper-based materials provision due to the lack of information about this solution (UNICEF, 2020).

The same report mentioned that 94% of the countries provided at least one educational response (yet it cannot be ignored that 6% did not provide any educational response to help students during the pandemic). Despite the positive news, researchers and educators lack data on the actual number of students reached by these emergency educational responses. The UNICEF (2020) report only provides data on the potential reach of such measures. These data were based on information gathered about the availability of technologies to study at home, and merit educational stakeholders' and researchers' attention. The report alerts that “at least 463 million—or 31 percent—of schoolchildren worldwide cannot be reached by digital and broadcast remote learning programs enacted to counter school closures” (UNICEF, 2020, p. 6). It is important to note that, due to the criteria chosen for data collection, this figure is clearly underestimated. For example, for statistical purposes, countries who were providing digital learning to the final year of elementary school, considered all elementary school students as being reached by this mode of delivering contents. Another interesting finding is that, globally, about 50% of the students who were attending elementary school by the time of school closure could not be reached by any alternative teaching mode. Considering these data, it is estimated that more than 215 million elementary school students were left behind without education during the school closure in 2020. In addition, a deep analysis allows the conclusion that the number of reached students varies between, but also within, countries. For example, data indicate that students from rural areas (even in developed countries) and low-income families were the most affected. The following quotation illustrates this proposition: “three out of four students who cannot be reached live in rural areas, but in low-income countries the percentage is even higher” (UNICEF, 2020, p. 11).

All of these data are disquieting and merit the attention of researchers and practitioners. For example, policy makers and educators could delve further into their analyses of the phenomenon by asking, “How many of the reached students actually used the provided educational solution(s) to help them learn and progress?” To the best of the authors' knowledge, there is currently no available data to answer this question, but the number of students benefiting from the alternatives modes of learning is likely to be significantly lower than 69%. In fact, the UNICEF report (2020) draws readers' attention to this issue. Even for students with access to technology at home, the number and type of devices available, the family environment, as well as the cultural values at home may limit the usage of devices (Moore et al., 2018; UNICEF, 2020; Xie and Yang, 2020). For example, during the period of confinement in 2020, parents teleworking and school-aged children studying in the same house with only one device available at home were likely to face difficulties to manage the usage of the device.

This alert is consistent with prior data. For example, the report by Moore et al. (2018), using data from a survey run in the United States, indicated that 14% of the participants (the majority being high school students from disadvantaged backgrounds) reported having only one device at home. Importantly, 56% of these students reported that this single device was a smartphone. Due to its characteristics, the usage of this type of device for school tasks (e.g., writing essays, completing complex project tasks) is limited (Moore et al., 2018). All considered, the potential reach of the educational responses through online remote learning could be significantly lower than that of which was disclosed in the UNICEF latest report (2020). In addition, constraints and obstacles of a social nature should be acknowledged while discussing educational responses through online remote learning, for example, parents, students, and teachers' low digital literacy, pressure on children and youth to do household chores (especially in the case of girls) or other types of work to help the family (e.g., cook family meals, help in the local shop), or lack of parental support in using the online or broadcast educational responses (UNICEF, 2020; Xie and Yang, 2020). Besides the external factors that may compromise students' learning opportunities, literature has been alerting to the role played by motivation and self-regulation on students' school progress (Zimmerman, 2002; Rosário et al., 2016). For example, students working remotely on their school tasks with limited support are still expected to sustain motivation and avoid potential distractors (e.g., online games, text messages), define learning goals and strategies to complete school tasks, organize the environment, and manage time and the available and very often scarce resources, as well as their negative emotions, such as the boredom associated with being at home and far away from friends (Brooks et al., 2020; Xie and Yang, 2020).

Learning and working remotely on school tasks requires displaying agency and self-regulatory skills (e.g., Raffaelli et al., 2018). However, as prior research has been alerting, not all students self-regulate their learning tasks efficaciously (Zimmerman, 2002; Vandevelde et al., 2015; Heirweg et al., 2019). Acknowledging the challenges posed by the COVID-19 pandemic during school closure, it appears pressingly more urgent to provide students with support in order to help them develop self-regulatory skills. For example, the Chinese Ministry of Education has recently alerted teachers to the need of providing students with guidance and support “to scientifically formulate home-based learning plans, rationally select resources, and focus on developing students' autonomous learning ability” (Zhou et al., 2020, p. 504).

All considered, the purpose of the current work is 3-fold: to (i) provide a description of a project aimed to promote self-regulated learning (SRL) competences using alternative modes of intervention (i.e., local radio broadcasting and letters) as a way to reach students, particularly those without digital resources; (ii) analyze its potential reach; and (iii) explore participants' perspectives about their experience during this project.

The current project is grounded on the need to equip children with SRL competences vital to respond to numerous challenges raised by the pandemic context. We believe that the social and educational relevance of this project is likely to help educators and school administrators improve students' SRL competences and progress.

### Theoretical Framework

Zimmerman model of SRL (2000) provides a relevant theoretical framework to support student independent remote learning, namely during the COVID-19 pandemic (e.g., Carter et al., 2020). This cyclical model considers the learning processes and motivational beliefs that occur before, during, and after a learning task, which correspond to three phases: forethought, performance, and self-reflection (Zimmerman, 2000, 2002). The forethought phase is comprised of two major aspects: (i) task analysis (where individuals define self-determined goals and a strategic plan to perform a task) and (ii) self-motivational beliefs, such as goal orientation (e.g., learning mastery-oriented), self-efficacy, outcome expectations, task interest, and task value (Zimmerman, 2000). Subsequently, the performance phase is comprised of two major aspects: (i) self-control (which includes the usage of different methods and strategies selected in the previous phase, such as self-instruction, imagery, attention focusing, and task strategies) and (ii) self-observation (e.g., overt and covert monitoring processes, such as self-recording and cognitive tracking of the task) (Zimmerman, 2000, 2002). Finally, the self-reflection phase encompasses: (i) self-judgment (e.g., self-evaluation of the final performance based on a criterion and attributing causal significance to the results) and (ii) self-reaction (e.g., self-satisfaction and adaptive or maladaptive reactions) (Zimmerman, 2000, 2002).

According to this model, each phase positively or negatively influences the following. According to the cyclical nature of Zimmerman's model, the last phase influences the subsequent forethought phase. As Zimmerman (2002) stresses in his model, these “self-regulatory processes are teachable” (p. 69) through direct instruction, modeling, guided practice, and feedback (Schunk and Zimmerman, 1997; Rosário et al., 2016; Quigley et al., 2018). Moreover, literature indicates that SRL enhances students' motivation and performance (Zimmerman, 2002; Mega et al., 2014). What is more, developing students' SRL is particularly important during school closure to prevent substantial academic losses (e.g., Azevedo et al., 2020).

### Purpose of Alternative Modes of Intervention Delivery During COVID-19 Pandemic

In the context of the COVID-19 pandemic, recent research has been focused on the impact on the psychological state of children, adolescents, college students, and adults (Li et al., 2020; Zhang et al., 2020; e.g., Golberstein et al., 2020). For example, findings have shown the presence of negative feelings, such as frustration and boredom, symptoms of stress, depression, anxiety, insomnia, or exhaustion in these populations (Brooks et al., 2020; Li et al., 2020; Zhang et al., 2020). However, individuals (including children) are not just products of what happens to them; they can mobilize available resources to build adaptive responses to new events or ongoing changes in the surrounding environment (Bandura, 2006, 2018), in this case, to COVID-19 pandemic. SRL competences refer to a set of processes that allow individuals to proactively and intentionally control how they approach the personal, behavioral, and environmental aspects, along with the consequent impacts on their behavior, in order to achieve specific self-set goals (e.g., Zimmerman, 2000, 2002; Rosário et al., 2019b).

Promoting SRL competences is even more important during COVID-19 school closure (Xie and Yang, 2020). Due to the closure of schools, students moved from the classroom to the living room, and were expected to continue learning, despite receiving little support from teachers. Moreover, during this period, many students had their caregivers working at home (e.g., housework, farming, teleworking) and the families were forced to compete for the available resources (e.g., internet spots, available devises, workspaces). These new and unexpected circumstances brought about important challenges for children and youth learning process, such as: the need to focus their attention on school tasks delivered online, follow classes or school meetings with poor internet connectivity or limited access, sustain motivation, or manage negative emotions while dealing with remote learning (Moore et al., 2018; UNICEF, 2020; Xie and Yang, 2020; Zaccoletti et al., 2020).

The self-regulated learning competences may be trained and consequently improved (e.g., Schunk and Zimmerman, 1997; Rosário et al., 2016; Azevedo et al., 2019). Grounded on a SRL framework (Zimmerman, 2000; Rosário et al., 2010), the research team designed various online (i.e., social media^1^, synchronous sessions, blog, videos, google forms) and offline responses. These tools were based on an evidence-based story-tool intervention (i.e., “Yellow Trials and Tribulations”; Rosário et al., 2007b), and were designed with the purpose of promoting SRL competences in school-aged children during school closure. The current work addresses the offline interventions designed to reach students with low digital literacy or those experiencing a lack of technological resources during the COVID-19 health emergency.

Firstly, a description of the original intervention is provided, followed by the procedures involved in the design of the project, with two modes of intervention delivery: (i) radio broadcasting and (ii) letters. In the results section, a description of the project, its potential reach, and the participants' perspectives of their experience during the project was provided. Authors believe that this work may equip students around the world, especially those with low digital literacy or access to technological resources, to deal with the challenges of studying at home. In addition, this research contributes to mitigate the negative impact of school closure on students' learning.

## Materials and Methods

### “Yellow Trials and Tribulations”: Description of the Original Intervention

Grounded on the social cognitive theory, which assumes that contextual variables and learning settings play important roles in students' motivation and self-regulation, the story-tool intervention, “Yellow Trials and Tribulations,” aims to promote SRL competences in children under 10 years old (Rosário et al., 2010). This story tells the adventures of the colors of the rainbow searching for their friend, Yellow, who disappeared in the woods (Rosário et al., 2007b). Anchored in the value that all people are important, and no one can be left behind, the colors put all their efforts to find their friend. As with any adventure, there are challenges and difficulties to overcome, and for the colors to be successful in their purpose, they have to learn and use SRL strategies. Children using this story tool will be encouraged to follow a metacognitive approach while reading the chapters through the characters' experiences, and by reflecting on the activities developed in each session of the intervention. Educators are expected to help children develop declarative (e.g., what is a plan), procedural (e.g., how to plan), and conditional (e.g., when and why they should plan) knowledge of the SRL processes and strategies learned (Rosário et al., 2010, 2017).

In this sense, characters model how to “think before, during, and after” acting, following the cyclical model PLEE—the acronym for PLanning, Execution, and Evaluation (Rosário et al., 2010, 2017), which is rooted in the SRL model by Zimmerman (2000). The PLEE model claims that the SRL is cyclical and adds a recursive loop to the process: each phase of the model is embedded within the whole sequential loop (Rosário et al., 2017). For example, regarding the planning phase, children are introduced to the concept of goal setting and are expected to learn how goals are defined, as well as design plans using strategies likely to help them achieve particular self-set goals. Children learn that in the following step, the execution phase, they are expected to put the plan into practice. To this aim, they are expected to monitor the action process, for example, by analyzing the (in)effectiveness of the strategy used to attain the goal set and making adjustments if necessary. Finally, children learn the importance of closing the first iteration of the SRL cycle by evaluating the final output. In this phase, children are encouraged to analyze if everything has gone as planned, if their efforts in doing tasks were successful, and reflect on the reasons for their success or failure (e.g., Rosário and Polydoro, 2014; Rosário et al., 2017). This evaluation informs a new planning phase, resuming the SRL cycle (Rosário et al., 2010). In sum, this project aims to promote children's autonomy and agency (i.e., set and sustain intentional actions toward a self-determined goal) in the learning process (Bandura, 2006; Rosário et al., 2010, 2017).

Prior to the pandemic, the intervention with this story tool was carried out in the classroom on a weekly basis, for ~60 min. Sessions of the intervention were structured as follows (Rosário et al., 2007a, 2010): setting the scene (i.e., creating the environment for the work routine; for example, students wear a yellow shirt and join together in the space created for this purpose), examining advance organizers (Ausubel, 1978) (i.e., reviewing prior events of the story and lessons learned), reading the chapter(s), along with exploration and reflection of the messages conveyed, and finally, a practical activity and take-home message. It is important to note that the reflection and discussion of the SRL processes and strategies conveyed in the chapters followed a microanalytic methodology and the three types of knowledge (Rosário et al., 2017). This methodology helps children develop strong knowledge about the various SRL strategies, and the opportunity to discuss how they can implement them, despite obstacles and hardships (Núñez et al., 2013; Rosário et al., 2017).

The intervention has been conducted with 4th graders from urban and rural contexts and has been shown to significantly enhance self-reported SRL competences and self-efficacy (Fernandes, 2009; Högemann, 2011, 2016), as well as observed behavioral (e.g., punctuality, class participation) and cognitive (e.g., asking for help, verbalizations related to the task planning) engagement in the classroom context (Rosário et al., 2016). Qualitative data gathered from post-intervention meetings with the teachers of the participating students showed positive results. For example, teachers enrolled mentioned that students had started using PLEE in their daily activities in contexts outside of the classroom (e.g., bake a cake at home, planning games in the playground of the school), showing knowledge transference (Rosário et al., 2019a). The vast corpus of research on this story tool has provided evidence of the benefits of this narrative-based intervention on students' SRL competences development.

### Intervention Setting Prior School Closure

Two educational stakeholders (i.e., Calouste Gulbenkian Foundation and Integrated and Innovative Plan to Combat School Failure of the Trás-os-Montes Intermunicipal Community) engaged in the promotion of students' socio-emotional skills, learning autonomy, and consequently their academic success through evidence-based interventions funded the current intervention. The research team, GUIA, from the School of Psychology at the Universidade do Minho was selected to set the evidence-based intervention, “Yellow Trials and Tribulations,” to promote SRL in elementary students from several schools across the country. According to the protocol of this research tool, the intervention must be implemented by a professional (e.g., teacher, psychologist, social educator) trained for this purpose by the research team. After each session, the implementer completes a session sheet to self-evaluate the session (e.g., protocol of the session compliance) and identifies difficulties to discuss with the research team. All session sheets are analyzed by the research team, and then followed up through a meeting with the implementers via videoconference to monitor the intervention implementation on a monthly basis. Eventually, the research team trained 59 professionals between May and December of 2019. Training lasted 50 h and followed a theoretical (e.g., motivation theories, SRL models) and a practical approach (e.g., simulation of a session). At the end of the training, in the 2019/2020 school year, the story-tool intervention was expected to be conducted at 11 schools from distinct urban and rural contexts in Portugal.

The ethics committee of the University of Minho, and Ministry of Education approved the intervention and its assessment protocol. The parents or caregivers of the students enrolled provided written informed consent to the distinct phases of the research (i.e., intervention, self-reports, interviews) in accordance with the Declaration of Helsinki.

Finally, a total of 1,103 students from 57 classes of the 3rd (typically 8 years old) (*n* = 550 students) and 4th (typically 9 years old) (*n* = 553 students) grades were enrolled in the intervention. By the time of school closure in Portugal (March 16, 2020), as a response to contain the spread of the COVID-19 pandemic, implementers had conducted a few sessions of the program (i.e., ranging from one to five sessions). In detail, the children enrolled had already read one up to five out of 17 chapters of the story-tool when the schools closed.

### Procedures for Alternative Intervention Development and Assessment

Due to the unexpected school closure, the research team started working on alternatives to continue delivering the program. The alternative intervention solutions needed to acknowledge and respond to the educational needs of the children without technological/online resources or with low digital literacy during school closure. To this aim, the research team followed two steps: (i) conducting a needs assessment, and (ii) setting a collaboration ground with the coordinators and implementers of the story-tool intervention.

The needs assessment was conducted through a video conference with the implementers of the intervention that took place 1-week post school closure. During this meeting, implementers reported (i) the challenges they were facing while teleworking (e.g., balancing teleworking and caregiving [this was particularly challenging for parents of very young children], difficulties to adjust educational support to children needs, and high workload); (ii) the responses taken by the school administrators to help families cope with the school closure (e.g., remote learning, school tasks delivered by e-mail, or printed in the school or the City Hall and delivered to the students' homes by police officers); (iv) data on the available resources and conditions of the participating children and families (e.g., no technological or online resources, parents working in farms or factories with limited ability to support children learning, limited time for children to use the computer due to parents' teleworking, and limited space at home to manage siblings' remote learning); and finally, (v) overload of school tasks). Some implementers reported that several City Halls were providing computer and internet connections to students from low socioeconomic backgrounds. This was an important resource for many families. Still, for those with low digital literacy, this was a social measure of limited use. Moreover, implementers declared their willingness to continue the intervention following alternative modes of delivery, and to further discuss the suggestions presented by the research team. Still, implementers were not able to decide by themselves, if, how, and when to follow through with the interventions interrupted. Teachers and parents were experiencing distressing situations with the school closure, and the school administrators had to evaluate the feasibility of continuing the project using alternative modes of delivery.

A few weeks later, the research team and the implementers met through videoconference to learn of their decision regarding the intervention, and to discuss possible solutions. Implementers and research team members agreed that potential solutions were needed to prevent leaving students behind and should be grounded in the core features of the original intervention “Yellow Trials and Tribulations.”

Eventually, in three of the 11 schools enrolled, the implementers decided not to offer offline modes of intervention, because these schools were already delivering contents via online modes. On the contrary, eight of the schools enrolled declared to be willing to continue the intervention following offline modes of delivery (six decided to deliver the intervention via offline and online modes, and two decided to deliver the intervention exclusively via offline modes). Finally, discussions between the research team and the implementers resulted in two alternative solutions, to deliver the intervention via: (i) radio broadcasting or (ii) letters. The former was suggested by one implementer who had connections with a local radio. The mode of delivery and the materials used to reach students were planned collaboratively between the research team and the implementers. The second mode of delivery was proposed by the research team, given that some schools already had someone responsible to deliver school materials by mail or in person. The school which had an implementer with connections to a local radio decided to use this mode of intervention delivery, whereas the remaining implementers decided to use the letters. Both modes are further described in the results section.

To assess these two modes of intervention delivery, the research team decided to use descriptive statistics of potential reach—the percentage of children eligible by the implementers to participate in the offline modes of intervention delivery. Eligible children were: (i) those without technological/online resources, or (ii) those recently granted internet connection (after school closure), or (iii) those identified by the implementers as having low digital literacy to enroll in the online mode of intervention delivery. Moreover, based on internet connection data provided by the implementers, the research team decided to estimate the number of children that would not be reached if no offline modes of intervention delivery were implemented. Additionally, the research team and the implementers discussed other methods to assess the project impact. The academic-community collaborative team was aware that the methods to collect data should not be an additional load to children and families already feeling overwhelmed. Finally, interviews with the participating students were the chosen method of data collection (see Data Collection subsection).

### Participants Selected for Interviews

In order to assess the project, 30 students of each mode of intervention were randomly selected to be interviewed. This number of students is expected to ensure enough diversity and the saturation of data patterns (Warren, 2002; Baker and Edwards, 2012). Parents of these students gave their consent to this interview.

Regarding the intervention through radio, four out of the 30 participants selected for the interview were excluded for different reasons (i.e., one did not pick up the phone call to schedule the interview and three parents influenced the child's answers during the interview). Unexpectedly, 18 children (nine girls) reported not to listen to the radio. Despite this, the research team decided to include them in the current study to further understand the phenomena (see Results section). In this group of children (Mage = 8.67; SD = 0.70), 13 were from the 3rd grade and five were from 4th grade. Finally, eight children (five girls) reported listening to the radio. In this group of children (Mage = 9.00; SD = 0.71), five were from the 3rd grade and three were from 4th grade. These participating students were from a deep rural context.

Regarding the intervention through letters, of the 30 participants selected for the interview, 14 were excluded due to two reasons (i.e., nine did not pick up the phone call to schedule the interview, and five parents influenced the child's answers during the interviews). In the end, 16 children (Mage = 9.21; SD = 0.58), four from the 3rd grade (two girls) and 12 from 4th grade (four girls), were interviewed. These 16 participants confirmed they had received and read the letters. The participating students were from a deep rural context.

### Data Collection Through Interviews

As previously mentioned, interviews were given in order to assess the two modes of delivery (radio and letters) of the project. The research team conducted the interviews with children who provided informed assent and whose parents provided informed consent. During the month of July, trained research assistants conducted interviews by phone call, video call, or videoconference (e.g., ZOOM), according to the participants' preference. Regarding the video call/video conference interviews, the recommendations of the literature (e.g., Irani, 2019) to ensure the privacy of the participants (e.g., use of headphones during the interview) were followed. Each interview lasted ~25 min.

The semi-structured interview script included open-ended questions addressing participants' experiences during the confinement period and home school (e.g., conditions of quarantine, resources, needs, and others), along with specific questions to assess their experiences during each mode of intervention delivery (Table 1). These questions were previously asked to three children in order to ensure comprehensibility. It is important to note that these three children did not participate in the study. No changes were made to the semi-structured interview script.

**Table 1:** Semi-structured interview script.

**Radio**	**Letters**
*Now, I would like you to tell me how following along with the story “Yellow Trials and Tribulations” went through the radio*.	*Now, I would like you to tell me how following along with the story “Yellow Trials and Tribulations” went as sent to you in the “Letters from the Rainbow.”*
*How was it for you to follow the colors adventure through the radio?*	*How was it for you to receive these letters?*
*How did you prepare yourself to listen to the chapters on the radio?*	*What do you think of the activities that were presented by the Colors of the Rainbow?*
*What activities did you do after reading the chapter(s) on the radio?*	–
*What did you learn?*	*What did you learn?*
*How can you use what you have learned on a daily basis? Please, give me some examples*.	*How can you use what you have learned on a daily basis? Please, give me some examples*.
*Imagine that you are now in the next year and that you are participating again in this project. What suggestions would you give to help children learn the lessons from the colors of the rainbow?*	*Imagine that you are now in the next year and that you are participating again in this project. What suggestions would you give to help children learn the lessons from the colors of the rainbow?*

### Data Analysis of the Interviews

Interviews transcriptions were independently analyzed by two research assistants using content analysis, following a deductive and inductive approach (Bardin, 1996). Regarding the deductive approach, prior to the analysis, a codebook was created based on the PLEE theoretical model (Rosário et al., 2010). Firstly, transcripts were read several times to get an overview of the data. Then, a deductive approach was used to code data, fitting data to the theoretical-driven codes (e.g., planning, executing, evaluating). The inductive approach was followed to identify new codes emerging from data (e.g., absence of teacher support, high workload). Then, data were aggregated, and final categories were selected (e.g., perceived learning, constraints). When discrepancies were found in the coded material, the researchers discussed them in order to reach a consensus. After this coding process, data were analyzed (e.g., categories frequencies), reorganized, and then interpreted. QSR International's NVivo10 was used to support all data analysis. Inter-rater agreement was calculated to ensure the precision of the coding scheme. The Kappa value was 0.91 and 0.86 for the interviews focused on the radio and letters, respectively; these values are considered “almost perfect” according to Landis and Koch (1977, p. 165).

## Results

This section addresses the results of the three purposes of the current work: (i) description of the two modes of intervention delivery (i.e., radio and letters), (ii) potential reach, and (iii) participants' perspectives about their experience throughout the intervention.

### Description of the Offline Intervention Modes of Delivery

#### Story Reading on the Radio and Paper Activity

This mode of delivery for the intervention involved the dramatized reading of chapters from the book, “Yellow trials and tribulations,” on the radio over the course of 6 weeks (every Tuesday, from 7:10 to 7:30 pm). In addition, a printed script (see Supplementary Figures 1–3) was sent to the participants through mail or via personal delivery (e.g., teacher). In this offline delivery mode, the script for each week included: the goal(s) for the week, advanced organizer (included after the first week of radio broadcasting), book excerpts and corresponding questions to prompt reflection, and finally a reminder of the following radio broadcasting session. To complement, the script included a consolidation activity designed to promote learning and strengthen the lessons learned (e.g., each activity was related to the chapters previously read). Moreover, families were invited to participate. Finally, the script ended with “today's lesson” question (e.g., “What was the most important thing you learned from this week's chapters?”). Table 2 illustrates the anatomy of the intervention, providing the goals for each week, the chapters and the activities proposed, as well as a brief description of each component.

**Table 2:** Anatomy of the adapted intervention using radio broadcasting: a week-by-week description of the goals, key components and related activities.

**S/W**	**Goals**	**Key components**	**Chapters/Activity**	**Brief description**
0	To explain and instruct families about the intervention (e.g., main steps).	Orientation guide	–	This guide describes the steps of the intervention. Participants receive an envelope to keep track of their activities during the intervention.
1	To analyze and anticipate consequences of the adopted behaviors. To value the role of effort and commitment in the learning process: Capacity vs. effort.	Reading	1, 2, 3, 4	The colors of the rainbow (the main characters of the story) are introduced. Yellow runs away from the rainbow and the other colors decided to start a quest in search of their friend. During this mission, they encounter characters that help them with valuable lessons.
		Activity	*Be a Teacher for 1 day*	Children are asked to imagine that they are teaching the characters of the story (e.g., the deer that only wanted to follow his will) and to write them three pieces of advice to help them overcome their difficulties.
2	To learn about the three phases of the self-regulatory process (planning, execution and evaluation—PLEE).	Reading	5, 6, 7^*^	The colors encounter new characters who teach them the PLEE model.
		Activity	*Make a colorful fruit salad*	Children are asked to make a colorful fruit salad with a family member, applying the PLEE process.
3	To analyze feelings and behaviors. To reflect on the consequences of behaviors over time (short, medium, and long term). To learn to control impulsivity, developing reflexivity.	Reading	8^*^, 9, 10	During their mission, the colors watch a group of *problems* having a picnic (e.g., lie, fear).
		Activity	*Emotions*	Children are invited to think on the *problem* that was more “present” throughout their own day, and on strategies to “smooth” it.
4	To identify the three phases of the PLEE process in stories and in everyday situations. To understand the concept *monitoring*.	Reading	11, 12	These chapters tell the story of “The Three Little Pigs.” The colors apply the PLEE model to the story.
		Activity	*Create a house, applying PLEE*	Children are asked to build a miniature house while applying the PLEE model and monitoring the process.
5	To understand the steps of the problem-solving process. To apply these steps to specific tasks.	Reading	13, 14	The Pirate Tree gave riddles to the Colors of the Rainbow.
		Activity	*Be a detective for 1 day*	Children are asked to play detective and help the Pirate Tree solve his problem.
6	To take responsibility for our own actions: the importance of error. To reflect on the lessons learned.	Reading	15, 16, 17	The colors found an injured bird and helped him.
		Activity	*Sentences and lessons to keep*	Children are asked to complete activities (e.g., select important sentences from the story that had stuck with them throughout the journey, and think what lessons they took from it).

*S/W, Sessions/weeks*.

* *In the original intervention, these chapters were not included. In this mode of intervention delivery, these chapters were read on the radio, but there were not included in the session activity (printed script of the session)*.

At the end of the program, children were invited to send all the material to their school/teacher. For that purpose, each received an envelope in the first week.

#### Letters From the Colors of the Rainbow

This mode of delivery for the intervention was developed through adaptations of the contents of the online and social media project, “COVID-19 in trials and tribulations” (see footnote 1) to an offline mode of delivery (i.e., letters). In order to make these materials visually attractive to children, all letters were stamped with the shape of a drop (inspired by the drops of the “COVID-19 Trials and Tribulations” project). Each drop was designed to be colored and cut out by the participant, so that by the end of the intervention, this collection of colored drops could form a rainbow (see Supplementary Figure 4).

In each letter, the protagonist was one of the colors of the Rainbow (e.g., Blue). In addition, every letter included three suggestions of activities promoted by the protagonist, each based on the following domains (see information in the footnote): (i) Learning something new (e.g., learn different drawing techniques); (ii) Collaboration at home (e.g., make the bed); (iii) Helping someone (e.g., call a friend to help with homework); or (iv) Leisure/Physical activity (e.g., different suggestions for physical exercise).

In addition to these activities and suggestions, other components were added to the letters intermittently. For example, the sections “solutions” and “week reflection.” The former provided the correct answers for the queries in the previous week's letter, allowing children to check their previous answers; and the latter, taken from the story, “Yellow trials and tribulations,” was developed to promote reflection on SRL important concepts (e.g., PLEE).

In sum, the new program, “Letters from the rainbow” was built to accompany children and their families until the end of the 3rd school term (the final term of the school year, typically, from April to mid-June). In total, the program consisted of a 12-week duration, and students were delivered 12 letters in addition to the welcome and the goodbye letters. The “Welcome Letter” aimed to explain to the families how the project works in this modality, and the “Goodbye Letter” was designed to be delivered at the conclusion of the program to thank all of the participants for their participation. Finally, “extra letters” termed “Tips from the Rainbow” were delivered occasionally, as a way to help children develop positive study strategies to apply in schoolwork during the 3rd term (e.g., how to build a timetable using PLEE), similar to the content published in the social media pages, “COVID-19 in trials and tribulations” on Facebook or Instagram.

For further details, Table 3 presents the goals and the anatomy of the intervention (detailed description of the letters and activities for each domain). Complementary to the letters, chapters of the book, “Yellow Trials and Tribulations” were previously sent to the children through mail or via personal delivery (e.g., teachers), so that participants could read the book autonomously.

**Table 3:** Anatomy of the intervention using letters: a week-by-week description of the goals, domains, and related activities.

**W/S**	**Goals**	**Letters**	**Domains**	**Brief Description**
1	To start the interaction with the children. To remember the characteristics of the colors. To explain and instruct families about the intervention (e.g., main steps).	Welcome letter	–	This is a welcome letter, where Yellow and the colors of the rainbow are introduced. The Letter explains how the intervention will work: what is expected, the weekly plan, etc.
	To promote physical activity and the application of PLEE. To promote logical thinking, problem-solving strategies, and the application of PLEE. To stimulate an organized physical space.	Letter 1	Leisure/Physical activity	*Basket and Socks*: The challenge is to throw the socks in the basket to gain muscle and try to hit as many socks in a basketball box.
			Learning something new	*Math trouble*
			Collaboration at home	Clean the living room (“Have you tidied up the room today?”). For example, fold the blankets or arrange the sofa cushions.
2	To promote logical thinking and problem-solving strategies, applying PLEE. To promote the knowledge of other forms of communication, and foster curiosity:	Letter 2**^a^**	Leisure/Physical activity	*Proverbmojis:* Discover what popular sayings are behind the emojis sequence.
	To promote communication with family or friends, even in difficult times.		Learning something new	*Sign Language:* Here is a picture of the alphabet of Portuguese sign language. The challenge is to try to form words.
	To evaluate and correct the previous activity.		Helping someone	*Call:* make a call to a friend or a family member and enjoy.
3	To promote the search for information using different sources.	Letter 3**^a^**	Learning something new	*Michelin star:* The challenge is to build a *recipe book*. Also, call a family member or friend to share favorite recipes.
	To promote physical activity. To promote interaction with the family. To reflect on the importance of organization (e.g., home, feelings).		Leisure/Physical activity	*Move it:* “What is your favorite exercise in physical education classes?”. Do this exercise and ask your family to do it with you.
	To evaluate/reflect on the consequences of lack of organization (e.g., school material, feelings). To foster awareness of what we feel and how it affects us.		Week reflection	“*There is a place for everything, and everything must be in its own place!”* (Lizard Stone, p. 12)
4	To promote organization. To provide a moment to relive past experiences and people. To promote interaction between family members.	Letter 4**^b^**	Collaboration at home	*Photoland:* Photographs are a way to relive the adventures we have been through! Take this time to revisit some photos and organize them by important dates or events.
	To promote physical activity. To foster interaction between family and neighbors. To help family and friends to do different activities.		Helping someone	*Partyland:* Choose a funny song, go to the balcony/window, and dance. Invite the neighbors to dance on their balconies too! it will be a Big Party.
	To promote planning and reflection on activities after quarantine. To promote interaction with the family. To foster creativity (e.g., decoration of the magic pot).		Learning something new	*Magical Pot:* (1) Choose a bottle and decorate it; (2) Take small papers and a pen; (3) Write post-quarantine goals and save them in the magical pot.
5	To create funny and interactive moments with the family. To foster creativity. To exercise thinking by guessing. To promote support by sharing a story. To promote interaction with other people (e.g., tell stories over the phone).	Letter 5	Leisure/Physical activity	*Mime game:* Play this game with the family (e.g., imitate animals, and ask other members to guess which animal you are imitating).
	To practice reading. To foster creativity—in the case of children who choose to tell a story of their own.		Helping someone	*Tell a story:* The challenge is to take your favorite book and read the story to someone of your choosing (even over the phone).
	To promote reflection about goals setting and the importance of defining a path to follow.		Week reflection	“*There is a way, there is always a way”* (Hiccups River, p. 15)
	To promote time management, considering all the activities students have (e.g., attending classes, studying, playing). To explain the purpose and the construction of a timetable, using PLEE.	Extra Letter 1	–	To complement the usual letters, this extra letter was developed to help children learn more about time management and how to be organized during their school tasks. In addition, by applying the PLEE model to this task, children learn to identify and understand the three phases of the PLEE process in everyday situations (e.g., “to build a timetable, we must plan, that is, think before what tasks we have…”).
6	To create awareness on the importance of recycling. To promote evaluation about current recycling practices. To promote recycling. To foster creativity.	Letter 6**^b^**	Collaboration at home	“*Do you recycle?”:* IF YES, then check if everything is done properly and leave reminders in each recycling bin informing about the materials and the conditions that should be put in the bin; IF NO, then it's time to start.
	To promote thinking.		Learning something new	*Animals in letters:* Challenge yourself in a word search puzzle
	To create fun and relaxing moments with the family.		Leisure/Physical activity	*Bowling:* Challenge your family to a Bowling game and learn who can knock down more pins. The pins can be bottles or other objects you find at home.
7	To encourage the reduction of water usage through planning creative strategies (helping the planet). To foster awareness on the importance of water.	Letter 7**^a^**	Helping someone	*Saving water:* Choose a 5-minute song and bathe while the music is playing!
	To promote thinking and creativity. To promote reflection on the importance of patience. To have the opportunity to evaluate and correct the previous activity.		Leisure/Physical activity	*Stop game:* It's a really fun game and helps to exercise your brain. Challenge the family to play! Together, pick a letter of the alphabet and have each player write a list of words for each categoric beginning with the chosen letter. The first player to write a word for all of the categories has to say “STOP.”
			Week reflection	“*Those who never give up will succeed”* (Hiccups River, p. 16)
8	To promote thinking. To create fun moments with the family. To promote logical thinking and practice.	Letter 8**^b^**	Learning something new	*Math Challenge:* Try to complete this multiplication table.
	To foster awareness on the importance of water.		Leisure	*Treasure Hunt:* Spend your energy by doing a treasure hunt with your family.
	To evaluate and find situations in which water is wasted.		Collaboration at home	*Dripping taps inspector:* Did you know that a tap dripping every 5 s for 24 h can waist 30 liters of water? The challenge is to reduce this number to zero! Inspect the taps of your house.
9	To recall positive moments from a new and challenging situation (e.g., social isolation). To promote creativity.	Letter 9	Helping someone	*Gifted Memories:* Exceptional moments deserve unrivaled memories! Choose a happy moment from the past few weeks and draw it!
	To promote the use of protective measures against the Coronavirus.		Collaboration at home	*Slowly you go far:* To walk you need to think ahead, be prepared, and be in a good physical condition! The challenge is to clean the soles of your shoes so that you can walk further and with style.
	To promote reflection on the importance of the agency, and the importance of others in this competence.		Week reflection	“*Learning more and better depends, above all, on what each one does”*(uncle *Jarbinhas*, p. 21)
10	To practice the content covered in classes and to promote thinking through a challenging activity that promotes student involvement.	Letter 10**^b^**	Learning something new	*Magical Square:* Complete the following math challenge.
	To promote planning skills while playing games. To promote fun moments with the family through the game.		Leisure/Physical activity	*Chinese Little Monkey:* Play this traditional game with the family.
	To help promote environmental awareness through the reuse of sheets of paper.		Helping someone	*Detective Paperwork:* How many unfinished notebooks or sheets written on one page do you have at home? Put all these sheets together and use them.
	To promote reflection and monitoring of daily feelings. To promote reflection on how different emotions influence the ways in which we behave and perform tasks.	Extra Letter 2	–	Designed as a complement, this letter was developed to help children to reflect further about daily feelings and emotions.
11	To encourage help among colleagues during school exercises. To practice asking for help (e.g., when having doubts in school).	Letter 11	Helping someone	*TeleProf*: Do you have questions about this week classes? Call a friend and see whether they can help you! P.S. you can do the opposite: help your friends!
	To promote communication with colleagues. To promote planning skills.		Leisure/Physical activity	*Hidden in the flour:* Ask someone to hide sweets in a bowl of flour, so that you can search for them using only your mouth, without your hands. But first think on your strategy.
	To promote fun moments, through the game. To promote reflection on the importance of initiative.		Week reflection	“*With closed wings, no one learns to fly!”* (Bird-Teacher, p. 21)
12	To promote logical thinking. To promote creativity.	Letter 12**^b^**	Learning something new	*Challenge of the Squirrel, Sarabico:* Find the mirror image of the following figure.
	To promote planning while doing outside activities (e.g., camping, party) in the home environment.		Helping someone	*Congratulations in time*: Create a calendar with all the months of the year and indicate the birthdays you want to remember.
			Collaboration at home	*Partyland*: How about having a party in a new place today? Create your own tent and have fun.
	To thank the children for their participation. To emphasize the importance their participation throughout the project. To summarize lessons learned. To encourage the importance of help others.	Goodbye letter	–	This was a goodbye letter, where the colors have the opportunity to thank the children for their participation.

*Children were provided the book Yellow's Trials and Tribulations to read autonomously*.

*S/W, Sessions/weeks*.

a *Solutions—This activity was added to the letters in specific weeks of the intervention. In this activity, solutions of the activities from the previous letter were presented to the children*.

b *The Week Reflection proposed by the research team was also added to the letters in specific weeks of the intervention*.

### Project Potential Reach

The project post-school closure was designed to include children from the 3rd and 4th grades with limited access to technological resources (e.g., computers, internet). These children were attending schools that had been implementing the original intervention “Yellow trials and tribulations” prior to the school closure due to the COVID-19 pandemic. From the 11 schools enrolled, eight declare their willingness to continue the intervention using offline modes of delivery.

**Figure 1:**
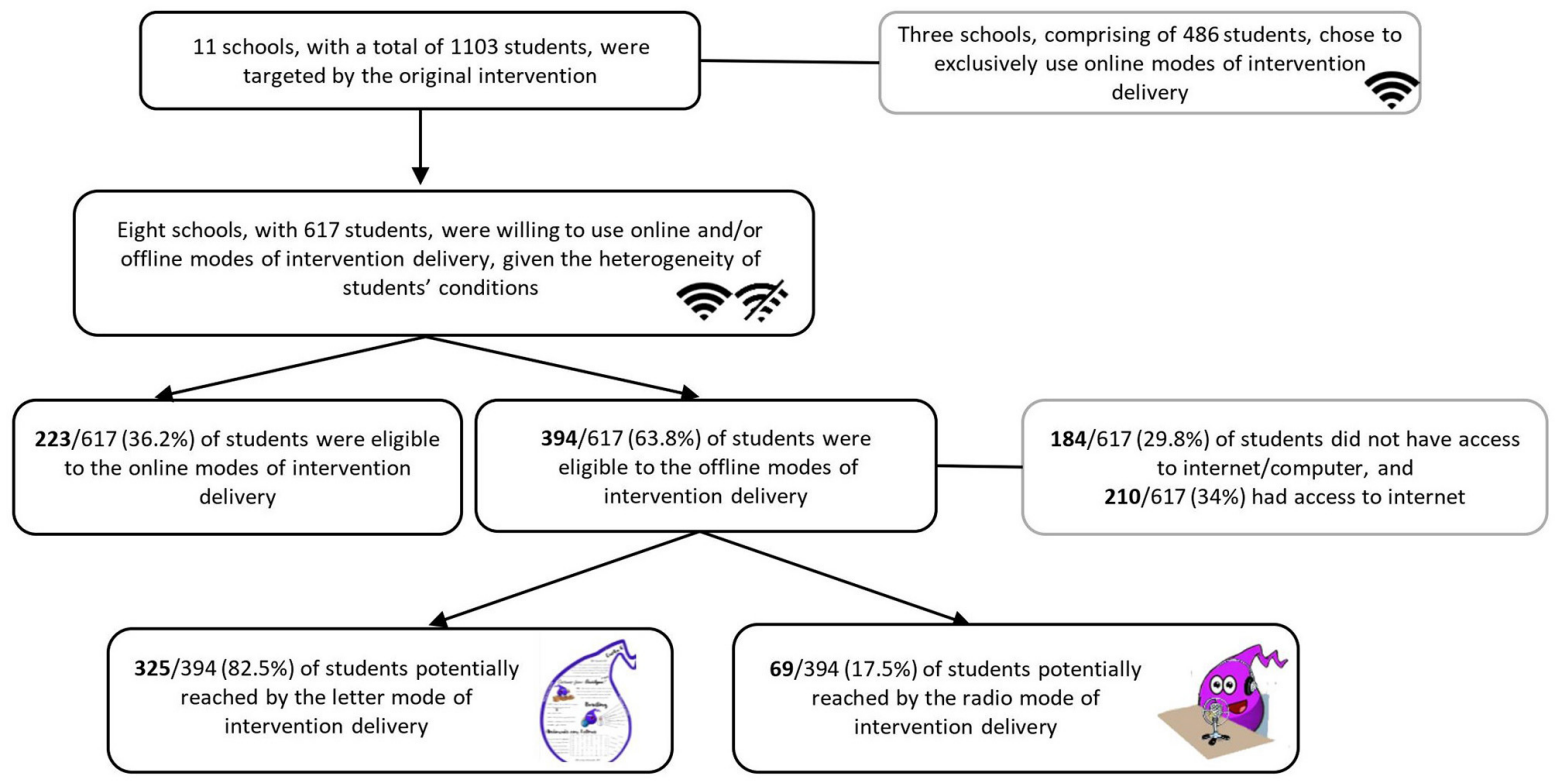
Flow chart of the potential reach of the two offline modes intervention delivery.

Figure 1 shows a flow chart presenting the potential reach of the program using radio broadcasting and the letters as modes of delivery. The eight schools enrolled included a total of 617 students. From this group, 394 participants were identified as having been offered one of the offline intervention modes of delivery. Based on these data, both modes of intervention delivery potentially reached 63.8% of students from the sampled schools. Figure 1 highlights that, from the 394 students, 184 (29.8%) had no computer or access to the internet, while 210 (34%) reported to have access to internet. This last result is due to three reasons: (i) the City Hall had recently provided internet access to the children (after school closure), so implementers decided to include them in the offline mode of intervention delivery; (ii) the implementers considered that the children did not have the digital competences to participate in the online modes of intervention delivery; and (iii) one implementer did not have technological resources to provide online sessions.

Table 4 presents an estimation of the children that would not reach in the case of non-existence of the offline modes of intervention delivery for each of the participating schools. Results show that the majority of the schools had a high percentage of children with no access to the internet. Specifically, five of the total eight schools had ~50% of children without technological resources. Moreover, it is important to note that one group of students enrolled in this project had access to the internet (School 2, see Table 4). In this particular case, despite these students having internet connection in their homes, the implementer was not able to provide online sessions. This school and its students accepted to enroll in this alternative intervention and participated until the end.

**Table 4:** Estimation of children not reached if the offline modes of intervention delivery were not implemented (by school), based on the number of students without internet access.

**Schools**	**Students eligible to the offline modes of intervention delivery (*n*)**	**Students without access to Internet (*n*)**	**Students without access to Internet (%)**
1^a^	39	20^*^	51.3
2^a^^,^ ^b^	66	0	–
3^a^	7	7^+^	46.7
4^a^	6	6	16.2
5^a^	9	9	8.3
6^a^	183	92^*^	50.3
7^a^	15	15	68.2
8^c^	69	35^*^	50.7

a *Letters as mode of intervention delivery*.

b *In this school, the implementer was not able to provide online sessions, despite the students having internet connection in their homes*.

c *Radio as mode of intervention delivery*.

* *In these schools, the number of participants without internet was not exact. Therefore, we estimated by 50%, the percentage of children without access to the internet*.

+ *In this case, participants reported no access to internet and computer*.

#### Story Reading on the Radio and Paper Activity

Assessment needs data showed that 69 (17.5%) children from the 3rd and 4th grades were potential participants in this delivery mode. In the beginning of this mode of intervention, the majority of the participants did not have access to the internet nor to other online resources, however, over the course of the program, the resources were provided to this group of children by the City Hall. Therefore, for this specific group of participants, the percentage of children not reached in the case of non-existence of this offline mode of intervention delivery was estimated, considering 50% of the cases without technological resources (*n* = 35 participants).

#### Letters From the Colors of the Rainbow

Assessment needs data showed that 325 (82.5%) children from seven schools were potential participants in this format delivery. In this group, about 37.8% of the students did not have access to the internet throughout the intervention. Two schools did not report data on children with no access to the internet/computer. In both schools, the percentage of children not reached in the case of non-existence of this offline mode of intervention delivery was estimated by reporting 50% of the cases.

### Qualitative Data Findings

This study explored children's perspectives of their experiences regarding the implementation of offline modes of the “Yellow Trials and Tribulations” intervention. The current section presents findings for both modes of delivery, radio broadcasting and letters. The frequency of occurrence criterion of the categories defined by Hill et al. (2005) was used to report results. Specifically, results from “variant” (i.e., more than 3 cases) categories are reported to provide diverse perspectives of the participating children. Throughout this research report, quotes from participants are presented to illustrate data.

#### Story Reading on the Radio and Paper Activity

When participants talked about their experience during the radio implemented intervention, four categories were identified in the analysis of the interviews: positive feedback, perceived learning, constraints, and suggestions. Children provided positive feedback about the possibility of continuing to listen to the story through the radio. Otherwise, as some mentioned promptly, they would not learn how the colors found their friend Yellow that run from the rainbow. Moreover, children considered the intervention through radio very creative, and different from the educational interventions they are used to. Another positive aspect of this mode was the involvement of the family. Children reported with joy that their parents or grandparents could listen to the story with them and help them with suggestions to complete the activities of the printed script.

When children were asked about the things they had learned with the “Yellow Trials and Tribulations” story, they majorly stressed their knowledge about “planning.” Participants were able to explain the meaning of this SRL strategy in their own words and provided some examples of its usage in their daily life, including doing homework or play. For example, one of the participants said “Planning is thinking before doing something, what we are going to need and what materials we are going to need, right? It is to think about what we are going to do and what objects we need” (R09). Regarding homework, participants explained that before starting doing it, they have to set aside time to complete the assignments, organize the materials needed, and plan their answers. Other participants exemplified how they applied this knowledge when playing, for example, hide-and-seek with friends: [when I play seeker] “First, I have to set a plan to find my friends. That is, I must observe what is around me, try to listen to every little noise, and then walk slowly without making noise until I find hidden spots. Then, when I find someone, I must run as fast as I can” (R07).

Other important information gathered from the interviews is related to the constraints perceived by the participants. Despite enjoying listening to the story through the radio, children reported to face a heavy workload due to school tasks, which prevented them from (fully) completing the activities suggested in the printed script. Often, participants stated, “we have a lot of homework to do; I do not have time for more tasks, I just listen to the story [through the radio]” (R03). Children's reports also mentioned the absence of teacher support to help them complete school tasks. For instance, this absence of support prevented the possibility to ask questions and for clarification of their doubts.

Moreover, the first questions of the interview script allowed to identify 18 children who did not know that the story was being broadcast by radio, and for that reason, they did not listen to the story. In fact, some parents did share with the research assistants who conducted the interviews that they believed the implementers had sent them the information previously. However, at that time, they must have missed that information, as the family was overwhelmed with work (e.g., farming, feeding the animals), information from the school, heavy workload due to extra school tasks, and laborious household management, as a result of all the family being at home. All these factors were perceived by these parents as strong barriers to their child's engagement in the project.

Finally, children were invited to provide suggestions to help children better learn the lessons of the story. The majority of the participants made the same suggestion justified with the same reason. Participants reported that it would be useful to include video calls in the program. This way, they would be able see and interact with their classmates while discussing the story, and ask questions to their teacher (i.e., implementer of the project). Other participants stated that it would be interesting to hear their classmates through the radio to learn with them.

#### Letters From the Colors of the Rainbow

When participants talked about their experience during the implementation of the intervention through the letters, four major categories were identified in the analysis of the interviews: positive feedback, perceived learning, constraints, and suggestions. Children said enthusiastically that the letters help them learn how to think through fun and creative activities. They added that they had really enjoyed completing the letters at home during quarantine. “Funny” was the most frequent word used by the participants to describe their experience when reading the letters and completing the activities.

When children were asked about what they have learned with the “letters of the Rainbow,” the majority responded: learning about emotions, learning PLEE, especially how to plan, and finally learning to never give up. Firstly, participants mentioned to have learned a variety of emotions, regarding how they feel, as well as how others feel, particularly when someone helps them. Secondly, children were able to recall the acronym, PLEE, that was taught by one of the characters of the story: “PLEE means planning, execution, and evaluation” (L01). The children provided examples of how to use PLEE in daily activities, including school tasks. For example, “we have to make a list of all the things that we have to do during the day [Planning]; then, when we are doing them [Execution], we have to check if we are doing them as planned, which means monitoring. In the end, we have to check if we have done everything in the list [Evaluation]” (L05). Other children detailed their understanding of the planning phase. Beyond providing its definition, they gave examples specifying aspects necessary to consider during the planning phase (“...we have to think when and for how long to do each type of activity, for example, when to study and when to play [with friends] or play sports…” L16). Some participants explained the usefulness of planning: “Usually people speak without thinking before…this happens to me sometimes... I'm talking about planning, I have to think before I say something, otherwise I end up saying things that I do not want to or should not say” (L15). Finally, children reported to have learned to never give up. They recalled one of the messages of the book “those who never give up will succeed” (Character Hiccups River, p. 16), which was sent in one of the letters (Letter 7). As one participant said, “if I want something, I have to keep doing it until I get there, and then, yes, I will get it” (L13).

Despite these positive findings, some participants reported to have read the letters, but not to have completed the activities suggested, due to the heavy workload of school tasks. This constraint was confirmed by the parents of these children at the end of the interview. Moreover, parents added that their children prioritized schoolwork. With many assignments, there was no time left for out-of-class activities, they said. In the opinion of these parents, the number of assignments already required for school was a strong barrier, and subsequently prevented their child from completing the activities.

Finally, when children were invited to provide suggestions to improve the project, the majority of the participants did not make any suggestions to improve this mode of intervention delivery. Some said, for example, “I would keep the project as it is” (L2). However, a few children reported the need for video calls to be included in the project to allow them to ask questions to the implementer.

## Discussion

The current work aimed to describe two offline modes of delivery of the “Yellow Trials and Tribulations” intervention during school closure due to the COVID-19 pandemic. Moreover, the potential reach of both modes, as well as participants' perspectives of their experiences during the project implementation were provided. Upon the closure of schools, and following the motto of the colors of the rainbow, “no one should be left behind,” the authors were concerned with reaching as many students as possible, especially those with low digital literacy or without access to technological resources. For this reason, researchers and implementers worked together to design alternative modes of reach based on the families' needs and available resources. From this partnership resulted two modes of intervention delivery: radio broadcasting and letters.

These two modes of delivery, the materials built, as well as the prior assessment needs, were developed in a very short period of time. Authors were committed to finding alternative modes of delivery for the “Yellow Trials and Tribulations” intervention as soon as possible (i.e., the radio broadcasting mode started 8 weeks after; and the letters mode 3 weeks after school closure). These efforts were important to help prepare children to deal with the challenges of studying at home and to lessen the negative impact school closure had on students' learning and well-being.

As in any other intervention, data sets helped to evaluate the project, and consequently provide opportunity for reflection about its strengths and limitations. In the current work, data on potential reach and participants' perspectives of their experience during the project implementation will be discussed. The global potential reach of the project (radio broadcasting and letters) was 63.8%. Of these, 30% were students without access to the internet. These data indicate that about 30% of the children previously enrolled would have been left behind without these alternatives modes of delivering the intervention “Yellow Trials and Tribulations.” This is a worrying figure, but it can be even higher for some of the schools enrolled (e.g., 68.2% in school 7). Globally, current findings are consistent with data from the recent survey conducted in several countries of the world reported by UNICEF (2020). Moreover, and consistent with the same UNICEF report, the percentage of students in the current work without access to internet may be underestimated. To this point, implementers collected data on students' internet connection accessibility at home, although, several factors may limit its usage, such as the number and type of devices available at home, the home workspace constraints, and the family cultural values (Moore et al., 2018; UNICEF, 2020; Xie and Yang, 2020). For example, families that declared to have internet connection access may not also have enough technological devices for the whole family, or their children may lack digital literacy. This could be the case of the children who, despite having internet connection, participated in this offline intervention (except the students from school 2, see Table 4). For this reason, in future interventions, implementers of the project may consider collecting more detailed information, not only about internet connection, but also of the number and type of devices available in the family. These data would have allowed the exact needs of children to be calculated (see Moore et al., 2018).

According to the UNICEF report (2020), radio broadcasting was the second most used remote learning option implemented worldwide during the pandemic, and the first option in low-income countries. Interestingly, paper-based materials were rarely used as a response during the pandemic (UNICEF, 2020). However, in the current work, the research-community collaborative team designed paper-based materials to be used in both modes of intervention delivery (i.e., session scripts and letters). This option does not require any family technological resources and may lead to a reduction in educational inequalities faced by children who are underserved or do not have the skills to use technological devices (e.g., Moore et al., 2018; Van de Werfhorst et al., 2020). Besides, extant literature indicates that paper-and-pencil assignments are as beneficial to students' academic engagement and performance as online responses (see Magalhães et al., 2020). All considered, educational stakeholders and practitioners could consider using paper-based materials as an educational response during the pandemic. Still, the authors recognize that this option was only possible due to the pre-existing strong community networks that allowed the delivery of materials to children on a weekly basis.

The interviews conducted with the children who participated in the intervention through radio and letters provided important insights about the project. In both groups, children made very positive comments regarding the continuity of the intervention in a different, creative, and funny way. The research team considers this finding very positive, because it transmits the message that despite school closure, the “Yellow” did not abandon them. Participating students could learn how to translate the SRL messages conveyed in the story-tool to life events. For example, the message conveyed: it is always possible to redefine the initial plan and execute it while overcoming obstacles. This general “take home” message is consistent with the theoretical framework grounding the intervention (see Zimmerman, 2002; Rosário et al., 2010). Moreover, during the COVID-19 confinement, it seems that particularly the letters, as they were perceived as “funny,” may have helped children to experience positive feelings (e.g., enjoyment) and well-being when completing the proposed activities at home. This is a very positive output, given that the particular context of pandemic is known to have a negative impact on the psychological state of people, including that of children (e.g., Golberstein et al., 2020; Li et al., 2020; Zhang et al., 2020).

Participants perceived learning in both modes of intervention with slight differences. Still, irrespective of the mode of delivery, students showed declarative knowledge about SRL processes and were able to provide examples of daily life applications (e.g., homework completion, playing with friends). In fact, students who master knowledge or skills in one domain are likely to apply that knowledge or skills in a different educational context (Salomon and Perkins, 1989). Findings indicated that children displayed several examples of knowledge transference (e.g., Perkins and Salomon, 2012) showing a robust domain of the SRL contents conveyed. This process of transfer of knowledge is the ultimate goal of the intervention (Rosário et al., 2010), and a hallmark of deep learning (Barnett and Ceci, 2002), because it represents the students' recognition of the relevance of those learning contents.

However, findings regarding radio broadcasting need to be interpreted with caution. As mentioned previously, implementers had conducted three to five sessions of the intervention by the time the schools closed. During these first sessions, children learned and discussed the process of planning. Thus, the current findings cannot be entirely allocated to the radio broadcasting mode of intervention delivery. Most possibly, radio broadcasting helped children recall previous knowledge. As some children reported, heavy workload prevented them from completing the activities suggested in the printed script, which may help to understand why their perceived learning was restricted to the content “planning.” In fact, just listening to the story is not a sufficient condition to promote SRL skills. As previously mentioned, in every face-to-face intervention session, the reflection and discussion of the SRL strategies embedded in the chapters is structured on the three types of knowledge and follows a microanalytic methodology (Núñez et al., 2013; Rosário et al., 2017). In addition, children are expected to complete a practical activity to consolidate the SRL training conveyed (Rosário et al., 2010). These two steps of the intervention sessions encourage children to reflect deeply about and train their uses of SRL strategies (e.g., be able to select the best suited strategies to complete particular tasks or overcome difficult circumstances) (Rosário et al., 2010, 2019a). In sum, beyond declarative knowledge, children also have to manage procedural and conditional knowledge of the SRL strategies and put them into practice with proximal guidance and feedback (Schunk and Zimmerman, 1997; Rosário et al., 2010, 2016). Based on current qualitative data, children did not have these opportunities, which are key requisites of the intervention success (Rosário et al., 2017).

As the children in the radio broadcasting group, part of the children in the letters group participated in some sessions prior to school closure. Interestingly, the last group of children reported several contents learned from the letters, such as the PLEE, various emotions, and to never give up (cf. Table 3). The letters are not limited to these contents, but these are interesting and important gains, considering that this delivery mode does not allow group discussions as face-to-face sessions do (one of the key requisites as previously mentioned). The activities proposed in the letters may have contributed to the reported positive findings, given that children were provided the opportunity to practice SRL strategies while doing the activities suggested (e.g., Letter 11, see Supplementary Figure 4). Notwithstanding, the group discussions, according to the microanalytic methodology, would have promoted children's deep reflection about SRL strategies (Rosário et al., 2016, 2017). Moreover, implementers' guidance and feedback prior to children's independent practice would be essential to enhance the accuracy of the SRL competences (Schunk and Zimmerman, 1997; Quigley et al., 2018). Despite the aforementioned limitations and the modest perceived learning reported by children, the positive findings provide support to the use of paper-based materials, in this case, letters, as a useful and successful alternative mode of intervention in the context of school closure during the COVID-19 pandemic.

To strengthen the benefits of the two offline modes of intervention, it is important to consider the constraints reported by the participating children. On one hand, the high amount of school tasks overwhelmed students and families in this new circumstance of their lives (i.e., studying at home with no proximal teacher support for a long period of time). This educational scenario is likely to have prevented: (i) some students from learning that the “Yellow Trials and Tribulations” intervention was being delivered through radio, and (ii) some students from being engaged in completing the suggested activities in the printed script. For example, in the case of letters, some students reported not to have completed the activities due to this reason. As literature indicates, participants' engagement in the intervention (e.g., completing the proposed assignments) influences its impact on reported SRL strategies (e.g., Azevedo et al., 2019). Hence, educators should consider calibrating the amount of work assigned to students, especially in this case of pandemic. In this situation, educators should not assign the amount and type of schoolwork similar to that assigned in regular circumstances (e.g., Zhou et al., 2020). On the other hand, participants mentioned the absence of teacher/implementer support as another important constraint. This is consistent with literature findings which stress limited support from teachers to prevent proximal guidance and feedback, two essential keys in the promotion of students SRL development as previously reported (e.g., Schunk and Zimmerman, 1997; Quigley et al., 2018).

Finally, the suggestions mentioned by the participants, namely in the radio broadcasting group, are aligned with the last constraint. The video calls^2^ would allow children to interact with their teacher and classmates. This interaction would facilitate asking questions, discussion, and deep reflection, which are important requisites to “Yellow Trials and Tribulations” intervention success (Rosário et al., 2016, 2017); as well as the promotion of emotional engagement (e.g., Havik and Westergård, 2020). The latter is of particular importance, given that emotional engagement predicts behavioral engagement (e.g., Skinner et al., 2009). In fact, studies have shown that students who are not emotionally engaged in school tasks are prone to be behavioral and cognitive disengaged (e.g., Symonds et al., 2016; Cunha et al., 2019). The loneliness of the radio broadcasting mode of intervention and the high workload of students may have compromised students' emotional engagement, and consequently their behavioral engagement in the intervention. This emotional aspect should be taken into account in the future offline modes of intervention in efforts to help maximize its benefits.

Despite the contributions of the current work to literature and practice, there are some limitations that require acknowledgment. The study was conducted in the context of a pandemic during school closure and addressed the educational needs of students with particular characteristics (i.e., participants from deep rural areas, some with limited technological resources). These factors prevent the generalization or replication of findings. Moreover, in three of the schools enrolled, the exact number of students with access to the internet was not provided (see Table 4). For this reason, we may have over or underestimated the percentage of children not reached if there was no offline mode of intervention delivery offered. In addition, the research-community collaborative team did not collect data about the participants' engagement in the project, which prevents calculation of the actual reach of both modes of intervention delivery. For this reason, we are not able to provide information about the participants who were not interviewed in each mode of intervention delivery. Future research needs to address this limitation and analyze the characteristics and contextual conditions of children not reached. Despite the efforts made by the research-community collaborative team to involve students in the offline modes of intervention delivery, through interviews, it was possible to identify children not reached through radio mode. Future implementation of the project needs to overcome this limitation. Finally, it is important to acknowledge the limitation related to interviews being the sole method of data collection. However, due to the particular context of this intervention during school closure, we believe that additional data collection (e.g., questionnaires to assess SRL) would have contributed to increasing children's and their families' overload. As previously mentioned, the research-community collaborative team aimed to avoid this situation.

## Conclusion

School closure in the context of COVID-19 pandemic brought notable challenges to children's learning with potential severe academic losses (e.g., Azevedo et al., 2020), not to mention the negative impact on their psychological state (e.g., Golberstein et al., 2020). In this scenario, SRL competences are key tools utilized to help children cope with these obstacles and adapt to the pandemic challenges (see Bandura, 2006, 2018). For this reason, the “Yellow Trials and Tribulations” intervention could not be completed by the time of school closure. Majorly, the extant educational responses are online followed by broadcast remote alternatives; however, there is data showing that more than 30% of children could not be reached throughout the pandemic through these modes (UNICEF, 2020). In this sense, it is essential to develop offline modes of intervention delivery beyond online format, in attempts to reach as many children as possible, especially, those without technological resources or with low digital literacy.

The current work focused on two offline modes of intervention delivery using radio broadcasting and letters, which may be of great interest to other contexts intending to promote SRL skills remotely. Of the 617 students enrolled in the eight schools, the offline modes of intervention delivery potentially reached about 64% of students; of these, 30% had no access to internet connection and 34% did. The two options described in the current work can also be useful to children who have technological resources and digital literacy. In fact, these modes can be an alternative, or even a complement, to help diminish excessive screen time through the computers or the use of television to support remote learning. This is a current concern due to excessive screen time with negative impacts on children's health (Vanderloo et al., 2020; Wong et al., 2020; e.g., Nagata et al., 2020). Current qualitative findings indicated that, despite some limitations (e.g., part of the children in the radio group was not reached; part of the children was not able to fully complete the activities suggested in the letters), children enjoyed the radio and letters modes of intervention delivery and were able to report what they had learned in the project: planning (radio group), learning about emotions, learning PLEE, and learning to never give up (letters group). These are positive findings, especially, in the context of the COVID-19 health emergency. In addition, data on the constraints, and suggestions are likely to help educators reflect on their practices during the pandemic, and eventually adjust them to respond to students' particular needs at this time.

To conclude, despite the challenges posed by the unexpected school closure due to the COVID-19 pandemic, the intervention “Yellow Trials and Tribulations” was implemented using different modes of delivery. Setting up this intervention was very challenging due to the constraints posed by the pandemic; still, promising positive results were found. Finally, this intervention stressed an important educational message: “no one should be left behind.” In fact, despite all obstacles and hardships, it was possible to readjust the plan set for the intervention and act in accordance with the new plans. As Bandura (2018) alerts, individuals are not just products of the circumstances (in this case, the pandemic), but rather agents that may control the events affecting their lives. Hopefully, by the end of the intervention, children will be more prepared to cope with the challenges of studying at home without proximal support from their teachers, and to display efforts to mitigate the potential academic losses due to school closure.

## Data Availability Statement

The datasets generated for this article are not readily available because interview transcripts are written in Portuguese. Requests to access the datasets should be directed to prosario@psi.uminho.pt.

## Ethics Statement

The studies involving human participants were reviewed and approved by Ethics committee of the University of Minho. Written informed consent to participate in this study was provided by the participants' legal guardian/next of kin.

## Author Contributions

JC and CS substantially contributed to the conception and the design of the paper, were responsible for the literature search, and wrote the manuscript with valuable inputs from AG, PS, CV, DL, and PR. Under PR supervision, AG, PS, CV, and DL were responsible for developing the intervention materials with community collaboration and for collecting data. CS analyzed quantitative data. JC, PS, CV, and DL analyzed qualitative data. PR was in charge of technical guidance and made important intellectual contribution in manuscript revision. All authors agreed for all aspects of the work and approved the version to be submitted.

## Conflict of Interest

The authors declare that the research was conducted in the absence of any commercial or financial relationships that could be construed as a potential conflict of interest.

## Footnotes

^1^An online response called “COVID-19 Trials and Tribulations” was developed and launched on Facebook® (Covid-19 em Sarilhos) and Instagram® (@covid19_em_sarilhos) 1 week after school closure. Chapters of the story “Yellow Trials and Tribulations” were shared with the followers in video format, every week. In addition, the project shared, every day, suggestions with activities sponsored by the colors of the rainbow. Activities were focused on four domains:

1. Learning something new—This domain was important to convey the message that, despite the school is closed due to the pandemic, the learning process continues (Zhou et al., 2020).
2. Collaboration at home—During the quarantine period, families had to reorganize/develop new household routines, requiring the collaboration of all family members. This domain was developed to respond to this reorganization and help keep home organized. Research has indicated the benefits of children collaboration in domestic chores, namely, in promoting their self-efficacy, satisfaction with life and prosocial behavior (e.g., White et al., 2019).
3. Helping someone—intends to instigate social responsibility in children during the pandemic period. Recent studies with children and adolescents have indicated higher social responsibility was associated to more social distancing behaviors; the latter are essential to fight the spread of the virus (e.g., Oosterhoff et al., 2020).
4. Leisure/Physical activity—this domain was developed to promote physical activity and leisure as a way to overcome the sedentary lifestyle that can result from the quarantine period (Dunton et al., 2020) and to stimulate the well-being and healthy development in children (Kuykendall et al., 2015; Freire et al., 2016).

^2^This suggestion indicates that part of the participating children have access to a smartphone with internet connection. This is consistent with the data reported by Moore et al. (2018) indicating that a part of disadvantaged background students has access to a single device, and this is a smartphone. Despite its limitations (e.g., single device shared by all family members with very limited internet connection), educators could consider using this resource for educational purposes whenever possible and for short periods of time given the limited internet connection through the smart phone.
