# Supplementary material for: No Children Should Be Left Behind During COVID-19 Pandemic: Description, Potential Reach, and Participants' Perspectives of a Project Through Radio and Letters to Promote Self-Regulatory Competences in Elementary School
Source: Front Psychol. 2021 May 5;12:647708. doi: 10.3389/fpsyg.2021.647708 (PMC8131506; doi:10.3389/fpsyg.2021.647708)
Supplement: Supplementary file 1 [file Data_Sheet_1.docx]

Supplementary Material

No Children Should Be Left Behind During COVID-19 Pandemic: Description, Potential Reach, and Participants’ Perspectives of a Project Through Radio and Letters to Promote Self-regulatory Competences in Elementary School

Jennifer Cunha^1^, Cátia Silva^1^, Ana Guimarães^1^, Patrícia Sousa^1^, Clara Vieira^1^, Dulce Lopes^1^, Pedro Rosário^1*^

^1^Unidade de Investigação Aprendizagem, Instrução e Carreira, Centro de Investigação em Psicologia, Departamento de Psicologia Aplicada, Escola de Psicologia, Universidade do Minho, Braga, Portugal

*** Correspondence:**Pedro Rosário
prosario@psi.uminho.pt

#
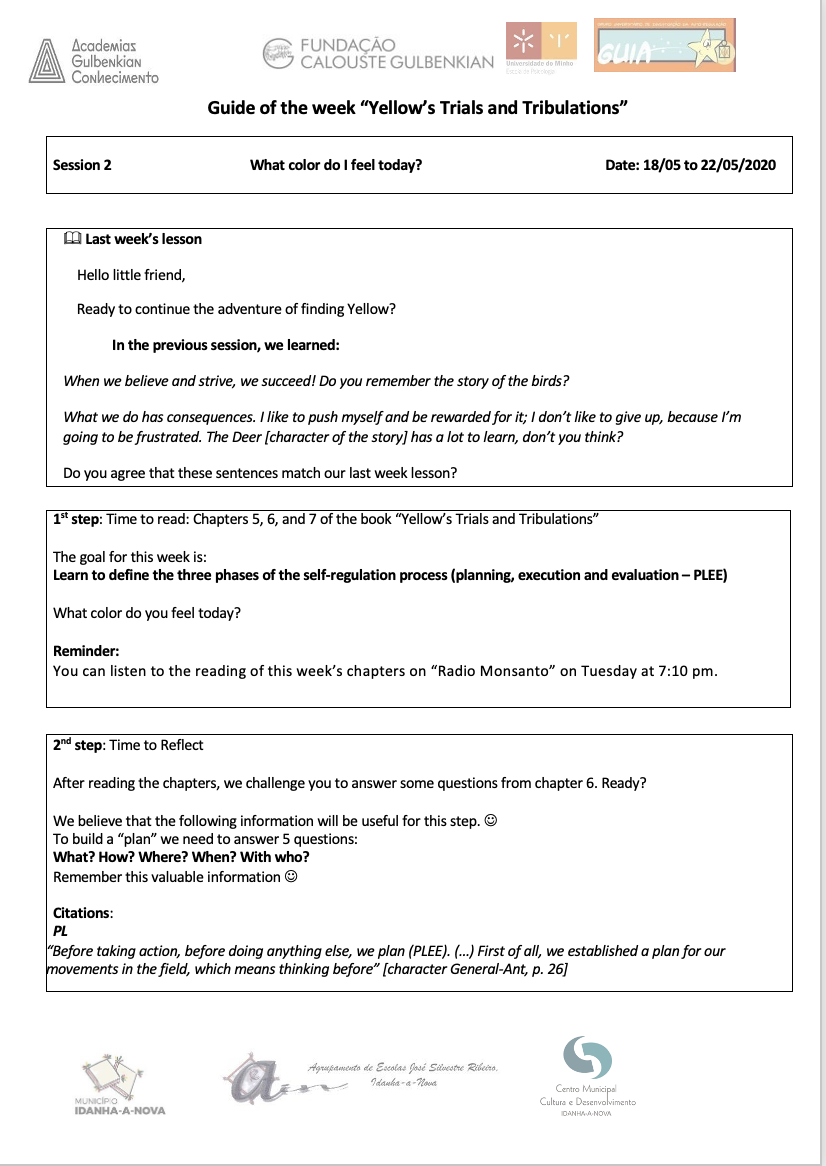
Supplementary Figures

# Supplementary Figure 1. Printed script of the radio broadcasting mode of intervention delivery – No. 2 (Front page).


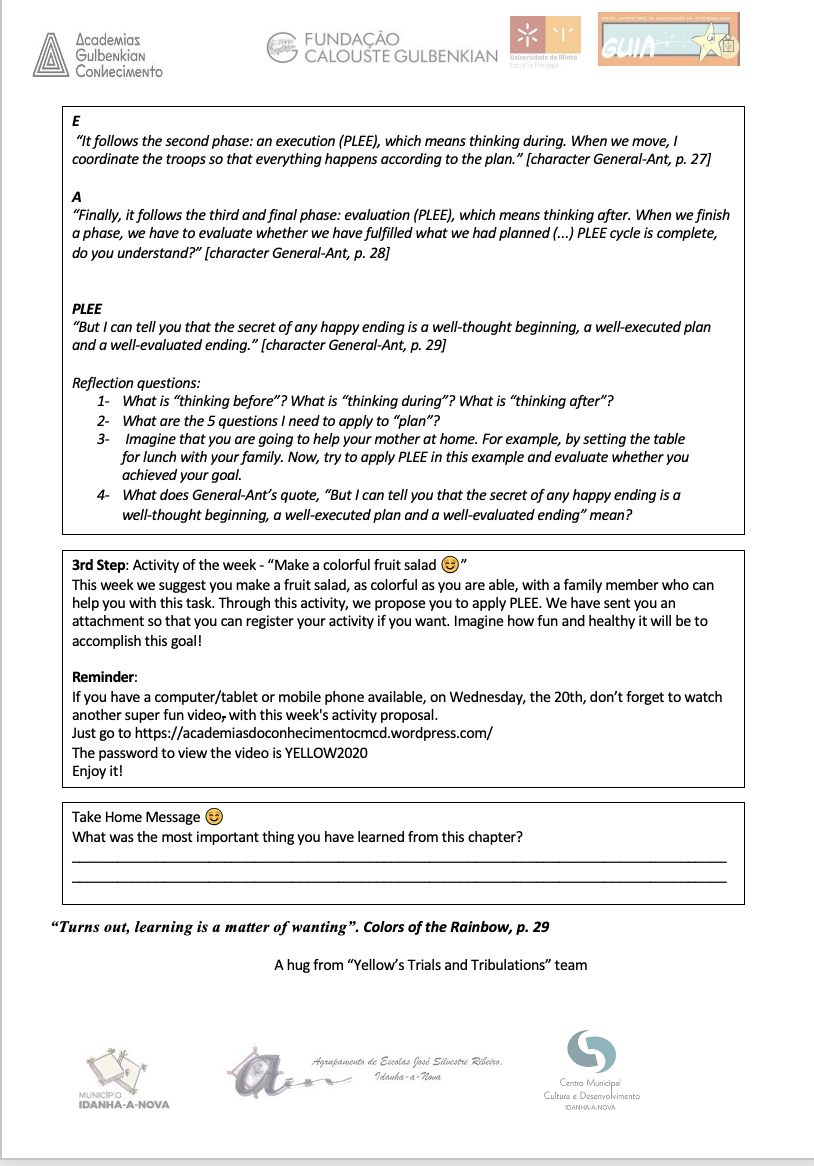


**Supplementary Figure 2.** Printed script of the radio broadcasting mode of intervention delivery – No. 2 (Back page).


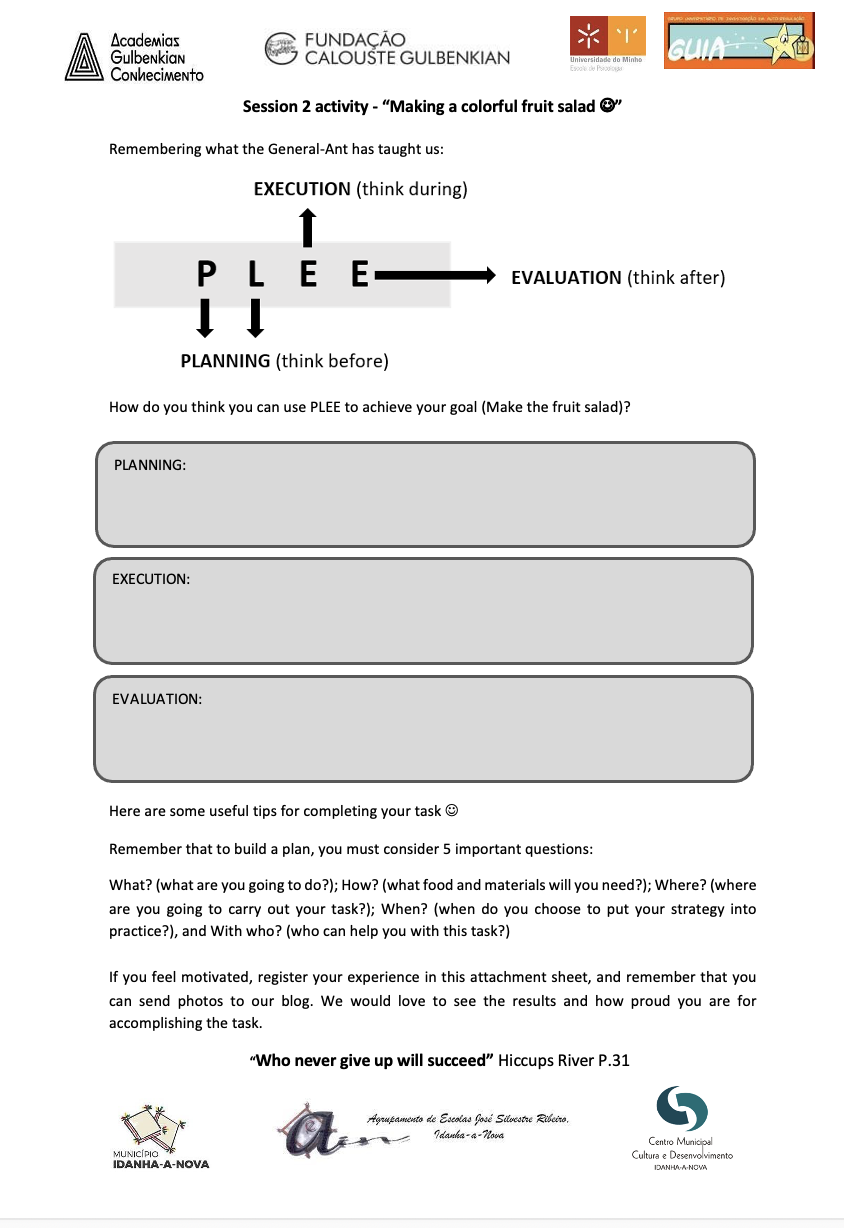


**Supplementary Figure 3.** Attachment of the printed script – No. 2.


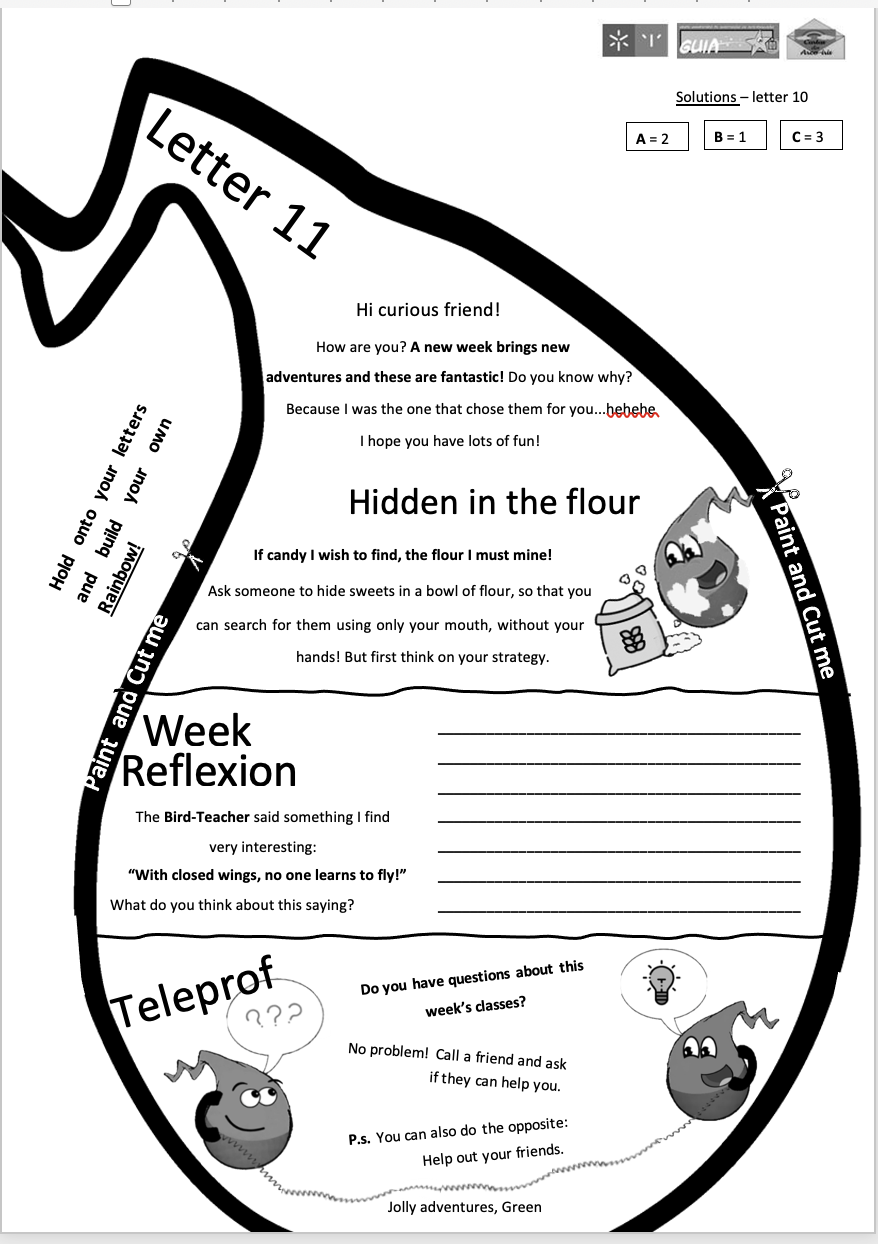


**Supplementary Figure 4.** Letter of the Colors of the Rainbow – No. 11
